# Involvement of the SIRT1/PGC-1α Signaling Pathway in Noise-Induced Hidden Hearing Loss

**DOI:** 10.3389/fphys.2022.798395

**Published:** 2022-05-10

**Authors:** Yu-Hui Liu, Yi-Hong Jiang, Cong-Cong Li, Xue-Min Chen, Li-Gui Huang, Min Zhang, Bai Ruan, Xiao-Cheng Wang

**Affiliations:** ^1^ Center of Clinical Aerospace Medicine, School of Aerospace Medicine, Key Laboratory of Aerospace Medicine of Ministry of Education, Air Force Medical University, Xi’an, China; ^2^ Department of Avation Medicine, Xi-Jing Hospital, Air Force Military Medical University, Xi’an, China; ^3^ Medical School of Chinese PLA, Beijing, China; ^4^ Senior Department of Otolaryngology-Head and Neck Surgery, The Sixth Medical Center, Chinese PLA General Hospital, Beijing, China; ^5^ National Clinical Research Center for Otolaryngologic Diseases, State Key Lab of Hearing Science, Ministry of Education, Beijing, China; ^6^ Beijing Key Lab of Hearing Impairment Prevention and Treatment, Beijing, China; ^7^ The 908th Hospital of Joint Logistics Support Force of PLA, Nanchang, China

**Keywords:** cochlea, noise-induced hidden hearing loss, ribbon synapse, oxidative stress, Sirtuin 1

## Abstract

**Objective:** To establish an animal model of noise-induced hidden hearing loss (NIHHL), evaluate the dynamic changes in cochlear ribbon synapses and cochlear hair cell morphology, and observe the involvement of the SIRT1/PGC-1α signaling pathway in NIHHL.

**Methods:** Male guinea pigs were randomly divided into three groups: control group, noise exposure group, and resveratrol treatment group. Each group was divided into five subgroups: the control group and 1 day, 1 week, 2 weeks, and 1 month post noise exposure groups. The experimental groups received noise stimulation at 105 dB SPL for 2 h. Hearing levels were examined by auditory brainstem response (ABR). Ribbon synapses were evaluated by inner ear basilar membrane preparation and immunofluorescence. The cochlear morphology was observed using scanning electron microscopy. Western blotting analysis and immunofluorescence was performed to assess the change of SIRT1/PGC-1α signaling. Levels of superoxide dismutase (SOD), malondialdehyde (MDA), catalase (CAT), ATP and SIRT1 activity were measured using commercial testing kits.

**Results:** In the noise exposure group, hearing threshold exhibited a temporary threshold shift (TTS), and amplitude of ABR wave I decreased irreversibly. Ribbon synapse density decreased after noise exposure, and the stereocilia were chaotic and then returned to normal. The expression and activity of SIRT1 and PGC-1α protein was lower than that in the control group. SOD, CAT and ATP were also influenced by noise exposure and were lower than those in the control group, but MDA showed no statistical differences compared with the control group. After resveratrol treatment, SIRT1 expression and activity showed a significant increase after noise exposure, compared with the noise exposure group. In parallel, the PGC-1α and antioxidant proteins were also significantly altered after noise exposure, compared with the noise exposure group. The damage to the ribbon synapses and the stereocilia were attenuated by resveratrol as well. More importantly, the auditory function, especially ABR wave I amplitudes, was also promoted in the resveratrol treatment group.

**Conclusion:** The SIRT1/PGC-1α signaling pathway and oxidative stress are involved in the pathogenesis of NIHHL and could be potential therapeutical targets in the future.

## Introduction

Hidden hearing loss (HHL), a recently reported auditory disorder, exhibits a normal hearing threshold, but leads to hearing problems such as tinnitus and hyperacusis, and influences the ability to understand speech in the presence of loud background noise (Bakay et al., 2018). For noise-induced HHL (NIHHL), the death of hair cells and spiral ganglion neurons (SGNs) are not the main cause, while synapse loss between inner hair cells (IHCs) and SGNs is the primary pathology, which is independent of both IHC and SGN loss (Kobel et al., 2017). No clinically applicable diagnostics or therapeutics for NIHHL have been approved yet (Kohrman et al., 2020), and the precise underlying molecular mechanisms remain unclear.

Ji (Ji et al., 2019) et al. applied metabolomics to explore the effects of noise on the inner ear of mice and reported the involvement of oxidative stress in NIHHL. Moreover, reactive oxygen species (ROS) are produced in the cochlea immediately after noise exposure, before any morphological damage emerges, and lasts for 7–10 days (Han et al., 2020). Sirtuin1 (SIRT1), an NAD-dependent histone deacetylase, plays a vital role in the redox system by deacetylating certain substrates, including class O of fork head box (FOXO), proliferator-activated receptor-gamma coactivator 1α (PGC-1α), p53, and Nrf2 (Nemoto et al., 2005; Maillet and Pervaiz, 2012; Mercken et al., 2014; Mitchell et al., 2014; Singh et al., 2018). PGC-1α is a transcriptional coactivator that regulates mitochondrial biogenesis and function, including oxidative phosphorylation and ROS detoxification (Rius-Pérez et al., 2020). Recent studies have reported that SIRT1 dysfunction is related to abnormal oxidative stress in cochlear cells and hearing loss (Kim et al., 2016; Xiong et al., 2017; Xiong et al., 2019; Affortit et al., 2021), and resveratrol treatment exhibits a protective effect against oxidative stress in cochlear hair cells by enhancing SIRT1 deacetylase activity (Xiong et al., 2019). In our previous study, we found that ginsenoside Rd ameliorated auditory cortex injury associated with military aviation noise-induced hearing loss (NIHL) by activating the SIRT1/PGC-1α signaling pathway (Chen et al., 2020). However, to date, the role and underlying mechanism of the SIRT1/PGC-1α signaling pathway in NIHHL have not been explored. We hypothesized that the SIRT1/PGC-1α signaling pathway is involved in pathophysiological changes in NIHHL. Therefore, we established animal models of NIHHL in guinea pigs to observe the dynamic changes in cochlear synapse and cochlear morphology and explore the involvement of the SIRT1/PGC-1α signaling pathway.

## Materials and Methods

### Animal Groups

One hundred fifty-nine male guinea pigs weighing approximately 250–300 g were purchased from the Experimental Animal Center of Air Force Medical University. None of the animals had a history of noise exposure and had normal hearing thresholds tested by auditory brainstem response (ABR). Under a 12 h light/dark cycle, all guinea pigs were routinely fed and provided with an adequate diet. Before the experiment, the animals were allowed 1 week to adapt to the new living conditions.

Guinea pigs were randomly assigned into 3 groups: a control group, which received no noise exposure but vehicle (6.67% DMSO in PBS); a noise exposure group (NE), which received noise exposure and vehicle (6.67% DMSO in PBS); a resveratrol treatment group (RES), which received noise exposure and orally administered with resveratrol (R5010, Sigma-Aldrich, United States) at the dose of 50 mg/kg body weight by using a gastric intubation once daily for 5 days until noise exposure.

The study was divided into two parts. In the first part (Table 1), 54 animals were used to observe the functional and morphological changes after a brief exposure to 105 dB SPL noise for 2 h. They were randomly assigned to the control group (n = 6), the noise exposure group (n = 24) or the resveratrol treatment group (n = 24). The 24 animals in the noise exposure group or the resveratrol treatment group were divided into four subgroups based on the time they were sacrificed for morphological analysis: 1day (1d), 1 week (1w), 2 weeks (2w), and 1 month (1m) post-noise exposure (PE) groups. The sample size was 6 in each subgroup. After hearing function was evaluated by ABR, one ear in each animal was used for IHC-SGN synaptic ribbons count and the other ear was used for SEM observation.

**TABLE 1:** Distribution of cochlear specimens of guinea pigs in the first part.

		Control Group	NE Group				RES Group			
Subgroup			1d PE	1w PE	2w PE	1m PE	1d PE	1w PE	2w PE	1m PE
Animal Number		6	6	6	6	6	6	6	6	6
Cochlear number	ABR	12	12	12	12	12	12	12	12	12
	synaptic ribbons count	6	6	6	6	6	6	6	6	6
	SEM observation	6	6	6	6	6	6	6	6	6

In the second part of the experiment (Table 2), 105 animals were used to observe the involvement of the SIRT1/PGC-1α signaling pathway in NIHHL. They were randomly assigned to the control group (n = 15), the noise exposure group (n = 45) or the resveratrol treatment group (n = 45). The 45 animals in the noise exposure group or the resveratrol treatment group were divided into three subgroups: 1d PE, 1w PE and 2w PE groups. The sample size was 15 in each subgroup. There were 30 cochlear specimens in each group. Six specimens were used for superoxide dismutase (SOD), malondialdehyde (MDA), and catalase (CAT) analysis. Six specimens were used for western blotting. Six specimens were used to detect the ATP. Six specimens were used to measure the SIRT1 activity. And six specimens were used for immunofluorescence.

**TABLE 2:** Distribution of cochlear specimens of guinea pigs in the second part.

		Control Group	NE Group			RES Group		
Subgroup			1d PE	1w PE	2w PE	1d PE	1w PE	2w PE
Animal Number		15	15	15	15	15	15	15
Cochlear number	SOD, MDA, CAT	6	6	6	6	6	6	6
	western blotting	6	6	6	6	6	6	6
	ATP	6	6	6	6	6	6	6
	SIRT1 activity	6	6	6	6	6	6	6
	immunofluorescence	6	6	6	6	6	6	6

All procedures were approved by the Institutional Animal Care and Use Committee of the Air Force Medical University in Xi’an, China.

### Noise Stimulation and Procedure

The broadband noise used in this experiment was environmental noise collected during the operation of a certain type of military helicopter in China’s aviation army, including engine noise and rotor noise, which were input to the loudspeaker (Soundtop SF-12, Jia-sheng Audio Equipment, China) for loop playback through a power amplifier (Soundtop QA-700, Jia-sheng Audio Equipment, China). The guinea pigs from the experimental groups were placed in a rat cage (approximately 11 cm × 11 cm×25 cm in size), and the speakers were placed on both sides of the cage. The noise intensity was measured using an A-weighted sound level (HCJYET HT8352, China) to ensure that the difference in the sound pressure level in the activity sphere of the guinea pigs was less than 3 dB SPL. The animals of the 1d PE, 1w PE, 2w PE, and 1m PE groups were exposed to 105 dB for 2 h. The control group did not receive noise stimulation, and the original cage background noise was *<*20 dB, while the other conditions were similar to those of the experimental groups.

### ABR Measurements

The guinea pigs of control, 1d PE, 1w PE, 2w PE, and 1m PE groups were subjected to ABR measurements (Otometrics, Taastrup, Denmark) at their respective points in time. Subsequently, they were anesthetized by i. p injection of 1% pentobarbital (0.3 ml/100 g). After anesthesia, they were moved into a soundproof room and placed on an electric blanket. The recording electrode was inserted subcutaneously into the middle of the vertical line between the ears on the head, the reference electrode was inserted subcutaneously behind the test ear, and the grounding electrode was inserted into the root of the right hind limb. The sound stimulus was composed of a 15-ms tone burst, with a rise-fall time of 1 ms at frequencies of 1, 2, 4, and 8 kHz. Brainstem auditory evoked responses, which were accumulated 600 times, were stimulated by density alternating short click sounds produced by the potentiometer. The lowest stimulation intensity that allowed the ABR III wave to be distinguished was considered as the auditory threshold. ABR wave I amplitudes were measured from the peak to the following trough.

### Tissue Preparation

In the first part, after the determination of ABR, the animals were sacrificed and the bilateral temporal bones of guinea pigs were removed, and the bilateral cochleae were separated immediately. The cochlear tissues used in the surface preparation of the basilar membrane were removed and then soaked in 4% paraformaldehyde, and those used for SEM were soaked in 2.5% glutaraldehyde. The specimens were perfused with the fixed fluid, transferred into the fixed solution overnight at 4°C and then used for surface preparation of the basilar membrane.

In the second part, the guinea pigs of control, 1d PE, 1w PE, and 2w PE groups were anesthetized by i. p injection of 1% pentobarbital (0.3 ml/100 g) at their respective points in time. The animals were sacrificed and the bilateral temporal bones of guinea pigs were removed, and the bilateral cochleae were separated immediately. The cochlear tissues used in section staining were fixed by 4% paraformaldehyde and then decalcified in 10% EDTA for 14 days. The decalcified cochlear tissues were embedded in paraffin to prepare 5 *μ*m sections for immunofluorescence staining. The remaining specimens were stored at −80°C.

### Cochlear Synapse Counting

The separated basilar membrane was immersed in 1% Triton X-100 (MP Biomedicals, United States) for 60 min, and then rinsed three times with PBS. After preincubation for 60 min at room temperature in ready-to-use normal goat serum (AR0009, BOSTER, China), the samples were incubated with rabbit anti-CtBP2 (1:200, ab128871, Abcam, United States) at 4°C for 24 h. The samples were then rinsed three times with PBS and incubated with the secondary antibody cy3-conjugated affinipure goat anti-rabbit IgG (1:200, SA00009-2, Proteintech, United States) for 2 h at room temperature. After incubation, the samples were washed three times with PBS. The basilar membrane was tiled on a glass slide and covered with a drop of antifade mounting medium with DAPI (P0131, Beyotime, China). The samples were observed using a confocal microscope (LSM 800, Zeiss, Germany). The number of CtBP2 positive spots was counted for each IHC. The regions were chosen per location (the basal, second, third, or apical turn) in each cochlea (six cochleae per group), and the total values of immunoreactive spots were counted in each region. The total number of spots for CtBP2 staining was divided by the total number of IHCs to obtain the average number of ribbons for each IHC.

### SEM Observation

The basilar membrane was immersed in fixative for TEM (G1102, Servicebio, China) overnight at 4°C. The samples were washed three times with PBS for 15 min each and then transferred into 1% OsO_4_ for 1–2 h at room temperature. Later, the samples were rinsed with PBS three times for 15 min each. The specimens were then dehydrated with 30, 50, 70, 80, 90, 95, and 100% gradient ethanol and isoamyl acetate. The samples were dried with a critical point dryer, attached to metallic stubs using carbon stickers, and sputter-coated with gold for 30 s. The results were observed using a scanning electron microscope (SU-8100, Hitachi, Japan).

Hair cells were counted using SEM. The regions were chosen per location (the basal, second, third, or apical turn) in each cochlea (six cochleae per group). Hair cells were considered to be absent if the stereo ciliary bundles and cuticular plates were missing.

### Western Blotting Analysis

Both cochleae of the same guinea pig were placed together in a tissue homogenizer. A 300 μl protein extraction reagent (78505, Thermo Scientific, United States) containing 3 mM PMSF was used as the protein lysate to extract the total cochlear proteins. Total protein concentration was measured using a BCA Protein Assay Kit (23250, Thermo Scientific, United States). A total of 30 *μ*g of each protein sample was denatured, separated on 4–12% Bis-Tris PAGE gels, and then transferred to polyvinylidene fluoride membranes (0.45 *μ*m, Millipore, Germany). The membranes were blocked in 5% fat-free milk powder for 2 h at room temperature at room temperature and were then incubated with rabbit polyclonal antibodies against SIRT1 (1:1,000, 13161-1-AP, Proteintech, United States), PGC-1α (1:1,000, A11971, ABclonal, China), p53 (1:1,000, A5761, ABclonal, China), or rabbit monoclonal antibody against ac-p53 (1:1,000, ab183544, abcam, United States), or mouse monoclonal antibody against β-actin (1:1,000, YM3028, Immunoway, United States) overnight at 4°C. After three washes with PBS containing 0.1% (w/v) Tween 20 (PBST) for 10 min each, the membranes were incubated in PBST with HRP-conjugated goat anti-rabbit IgG (1:10000, CW0103S, Cwbio, China) or HRP-conjugated goat anti-mouse IgG (1:10000, SA00001-1, Proteintech, United States) for 1 h at room temperature and were detected using enhanced chemiluminescence detection reagents (Millipore, United States). The band was obtained and analyzed using the FUSION FX SPECTRA (Vilber, France).

### Detection of Superoxide Dismutase, Malondialdehyde, Catalase and ATP Levels

Both cochleae of the same guinea pig were placed in the same EP tube, and the samples were prepared with 0.9% normal saline as a 10% homogenate. SOD assay kit (WST-1 method, A001-3, Jiancheng Biotechnology, China), MDA assay kit (TBA method, A003-1, Jiancheng Biotechnology, China), CAT assay kit (Visible light, A007-1, Jiancheng Biotechnology, China) and ATP assay kit (Phosphomolybdic acid colorimetric method, A095-1-1, Jiancheng Biotechnology, China) were used according to the manufacturer’s instructions. The chromaticity of each group was monitored using a microplate reader (BioTek, United States) at 450 nm for SOD activity, 532 nm for MDA levels, 405 nm for CAT levels and 636 nm for ATP levels.

### Detection of SIRT1 Activity

Both cochleae of the same guinea pig were placed together in a tissue homogenizer. A 300 μl protein extraction reagent (78505, Thermo Scientific, United States) was used as the protein lysate to extract the total cochlear proteins. Total protein concentration was measured using a BCA Protein Assay Kit (23250, Thermo Scientific, United States). NAD-dependent deacetylase activity was measured using the SIRT1 Assay Kit (ab156065, abcam, MA, United States) according to the manufacturer’s instructions. Fluorescence intensity was read continuously for 30 min at 2-min intervals with excitation at 340 nm and emission at 460 nm using a microplate reader (BioTek, United States).

### Immunofluorescence

Cochlea sections were deparaffinized and hydrated, and then rinsed three times with PBS. The sections were immersed in 1% Triton X-100 (MP Biomedicals, United States) for 10 min, and then rinsed three times with PBS. After preincubation for 60 min at room temperature in ready-to-use normal goat serum (AR0009, BOSTER, China), cochlea sections were incubated overnight at 4°C with rabbit polyclonal antibodies against SIRT1 (1:100, 13161-1-AP, Proteintech, United States), PGC-1α (1:100, A11971, ABclonal, China) separately. After three washes, sections were incubated with cy3-labeled goat anti-rabbit IgG (for SIRT1, 1:200, SA00009-2, Proteintech, United States) and FITC-labeled goat anti-rabbit IgG (for PGC-1α, 1:200, EK023, Zhuangzhi Biotechnology, China) at room temperature for 2 h and counterstained with DAPI. A confocal microscope (LSM 800, Zeiss, Germany) was used for observation.

### Statistical Analysis

The results are presented as the mean ± SEM. The hearing threshold, ABR wave I amplitude, immunofluorescence staining results, protein expression, SOD, MDA, CAT and ATP activities, and SIRT1 activity were statistically analyzed using one-way ANOVA. Dunnett test was used to compare the experimental and control groups. Differences were considered significant at *p* < 0.05. Statistical analyses were performed using GraphPad Prism 7.0 (GraphPad Software, United States) and SPSS 23.0 (IBM Corporation, United States).

## Results

### Noise Exposure Induces Noise-Induced Hidden Hearing Loss in Guinea Pigs and Resveratrol Promotes the Recovery of Auditory Function in Noise-Induced Hidden Hearing Loss Model

**FIGURE 1:**
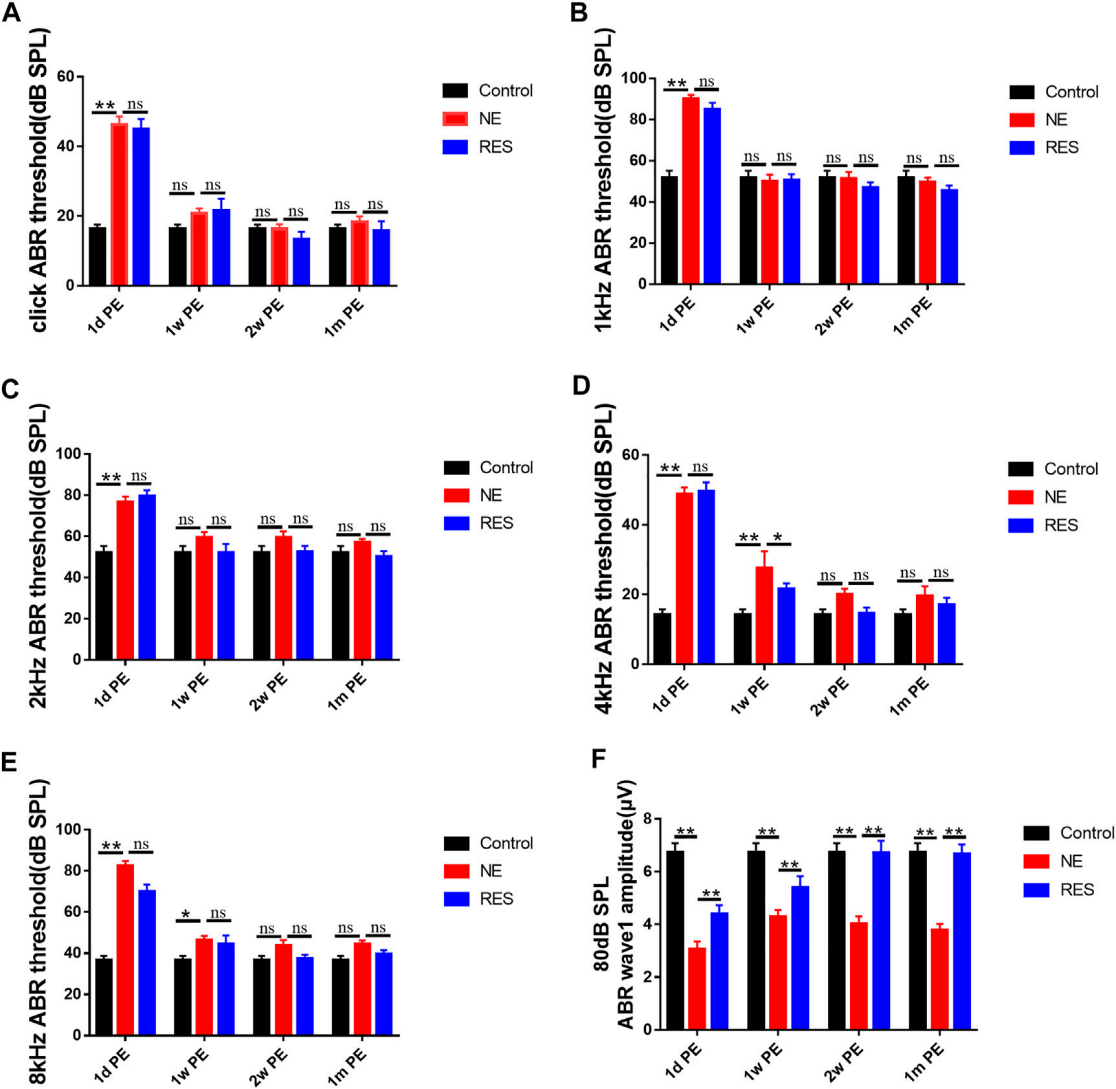
ABR after noise exposure, the values are presented as the means ± SEM. **(A)–(E)** The alterations of ABR threshold of guinea pigs in each group after 105dB SPL military helicopter noise exposure. **(F)** The alterations of 80 dB SPL ABR wave I amplitude of guinea pigs in each group after 105dB SPL military helicopter noise exposure (µV). Note: n = 12 ears from 6 animals in each group. *p < 0.05, **p < 0.01.

First, to observe the auditory function after noise exposure, we measured the ABR threshold values (Figures 1A–E). In the NE group, the results showed a temporary threshold shift (TTS) in guinea pigs after noise exposure. Compared with those in the control group, the average ABR threshold values were increased significantly 30–40 dB at all frequencies at 1d PE (*p* < 0.01). With the extension of time, the hearing threshold of guinea pigs recovered gradually. The average ABR threshold values returned to the normal level at 2w PE (*p* > 0.05). The ABR threshold values in the RES group exhibited the same tendency. The recovery of ABR threshold in the RES group is earlier than that in the NE group.

Next, the changes of ABR wave I amplitude were measured. In the NE group (Figure 1F, Supplementary Figure S1), supra-threshold amplitudes of wave I of the ABR (in response to clicks) showed a significant reduction at 1d PE (*p* < 0.01), and it remained lower at 1m PE, compared with the control group (*p* < 0.01). In the RES group (Figure 1F, Supplementary Figure S1), wave I of the ABR showed a significant increase (*p* < 0.01), compared with the NE group.

### Noise-Induced Hidden Hearing Loss Manifests a Loss of Ribbon Synapses Which Could Be Restored by Resveratrol

**FIGURE 2:**
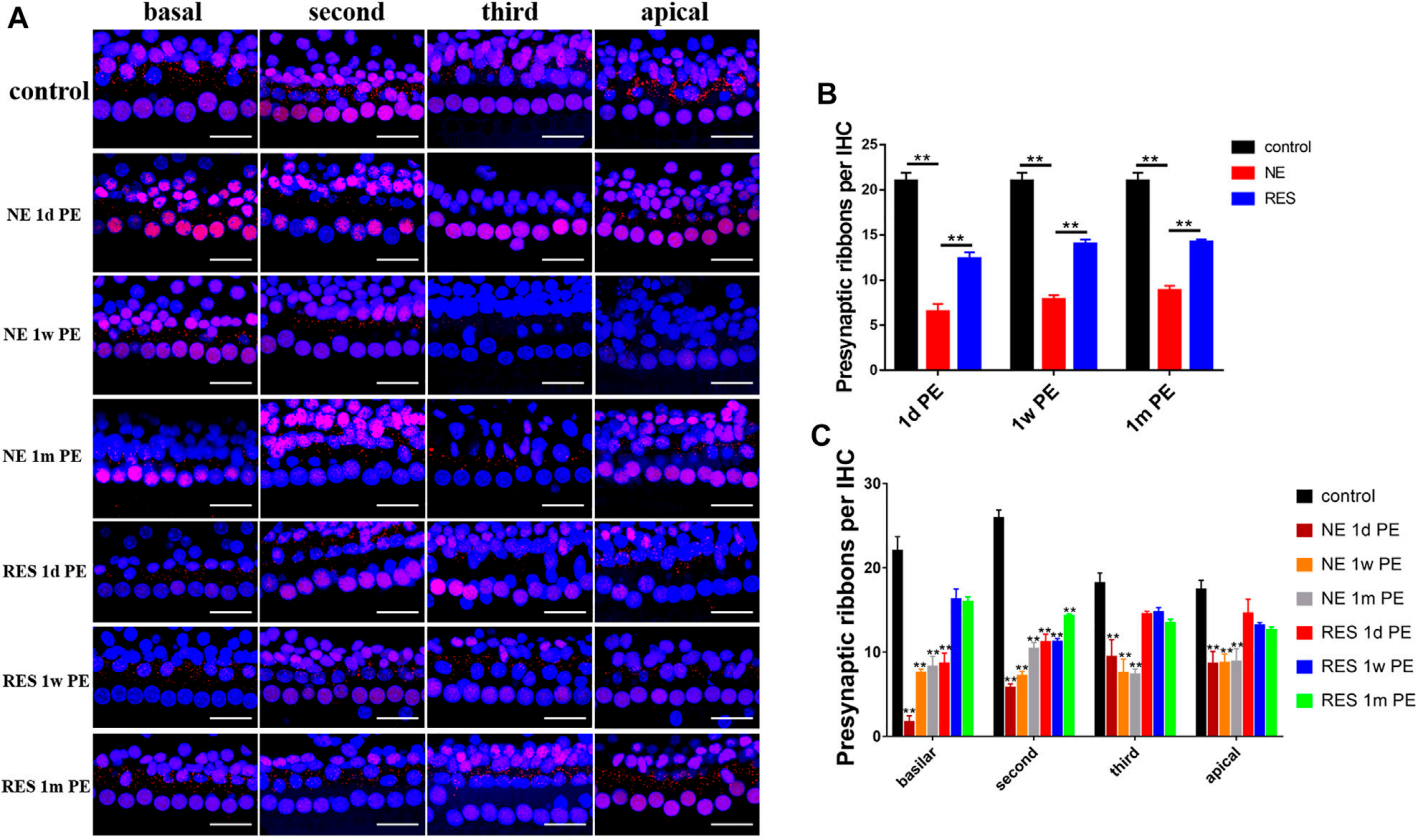
Noise induced changes in ribbon density (ribbons per IHC), the values are presented as the means ± SEM. **(A)** Representative images showing ribbon changes by the noise exposure. Scale bar = 100pixel. **(B)** The ribbon density at different time after noise. n = 24 locations in each group. **(C)** Ribbon changes of different turn by the noise exposure. n = 6 in each group. Note: In **(B)**, *p < 0.05, **p < 0.01, as compared with NE group. In **(C)**, *p < 0.05, **p < 0.01, as compared with control group.

To explore the effect of noise exposure on the ribbon synapses, we observed the changes of the number of ribbon synapses (Figure 2). In the NE group (Figure 2B), the averaged ribbon number for each IHC was 19.68 ± 0.73 in the control group and reduced to 6.51 ± 0.84 at 1d PE (reduced to 31.03% of the control). A partial recovery was observed later at 1w PE (7.86 ± 0.45, 37.45% of the control) and 1m PE (8.85 ± 0.51, 42.13% of the control). The ribbon density of every experimental group was significantly lower than that of the control group (*p* < 0.01). In the RES group (Figure 2B), the averaged ribbon number for each IHC reduced to 58.86% of the control group at 1d PE, and a partial recovery was observed later at 1w PE (66.62% of the control) and 1m PE (67.59% of the control). Figure 2B presents that the synaptic counts for the RES group were significantly increased at 1d PE, 1w PE and 1m PE, compared with the NE group, suggesting that resveratrol promotes the recovery of synapses.

In the NE group (Figure 2C), a reduction in ribbon density was observed across the whole cochlear region, but was more distinct in the high-frequency region. In the RES group (Figure 2C), the reduction in ribbon density was only observed in the basilar turn and the second turn at 1d PE, and the ribbon density in the basilar turn returned to the normal level at 1m PE.

### Stereocilia Damage Is Reversible in the Noise-Induced Hidden Hearing Loss Model and the Damage Could Be Attenuated by Resveratrol

**FIGURE 3:**
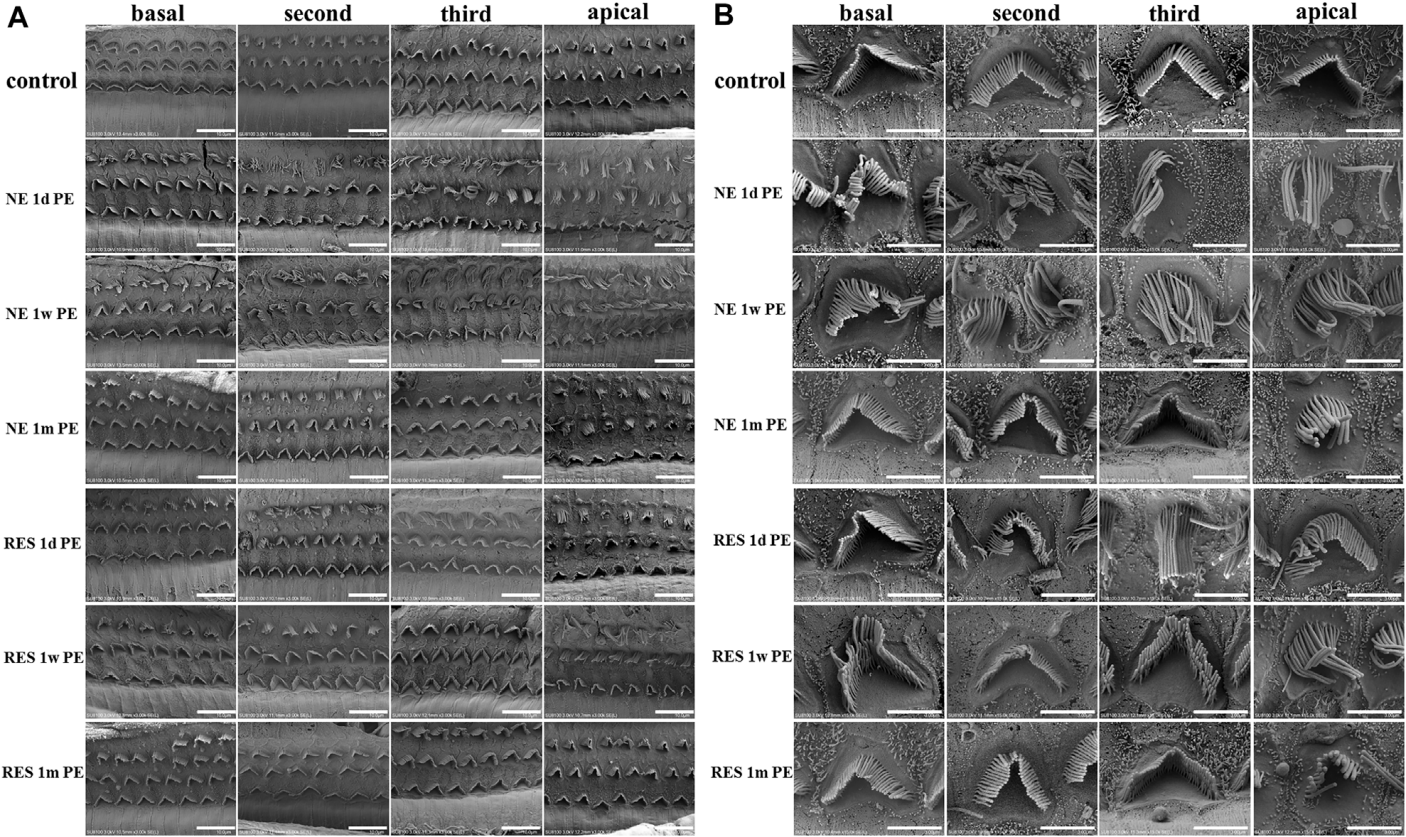
Noise induced changes of the morphology of basilar membrane after noise exposure. In **(A)**, scale bar = 10 µm. In **(B)**, scale bar = 3 µm.

To explore the morphology of cochlear hair cells, including stereocilia, we used SEM to examine the variation in cochlear hair cell structure. In the control group (Figure 3), three intact rows of mechanosensory hair cells were observed on the basilar membrane. The OHCs were neatly arranged, and the cilia were clearly visible and arranged in “V” shape. In the NE group, the arrangement of the OHCs was chaotic, with obvious lodging and fusion at 1d PE and 1w PE. Among them, the third layer of OHCs was the most severely damaged. Compared with the 1d PE group, the cilia of OHCs were more disordered in the 1w PE group. With time, the stereocilia of OHCs mostly returned to normal in the 1m PE group, but the cilia in the partial region still exhibited disorder. In the RES group, the disorder was less severe, compared with the NE group. And in the 1m PE group, the cilia exhibited no disorder.

The count of hair cells stayed almost intact until 1 month post noise exposure without significant loss (Supplementary Figure S2).

### SIRT1/PGC-1α Signaling Pathway Plays a Role in Noise-Induced Hidden Hearing Loss

**FIGURE 4:**
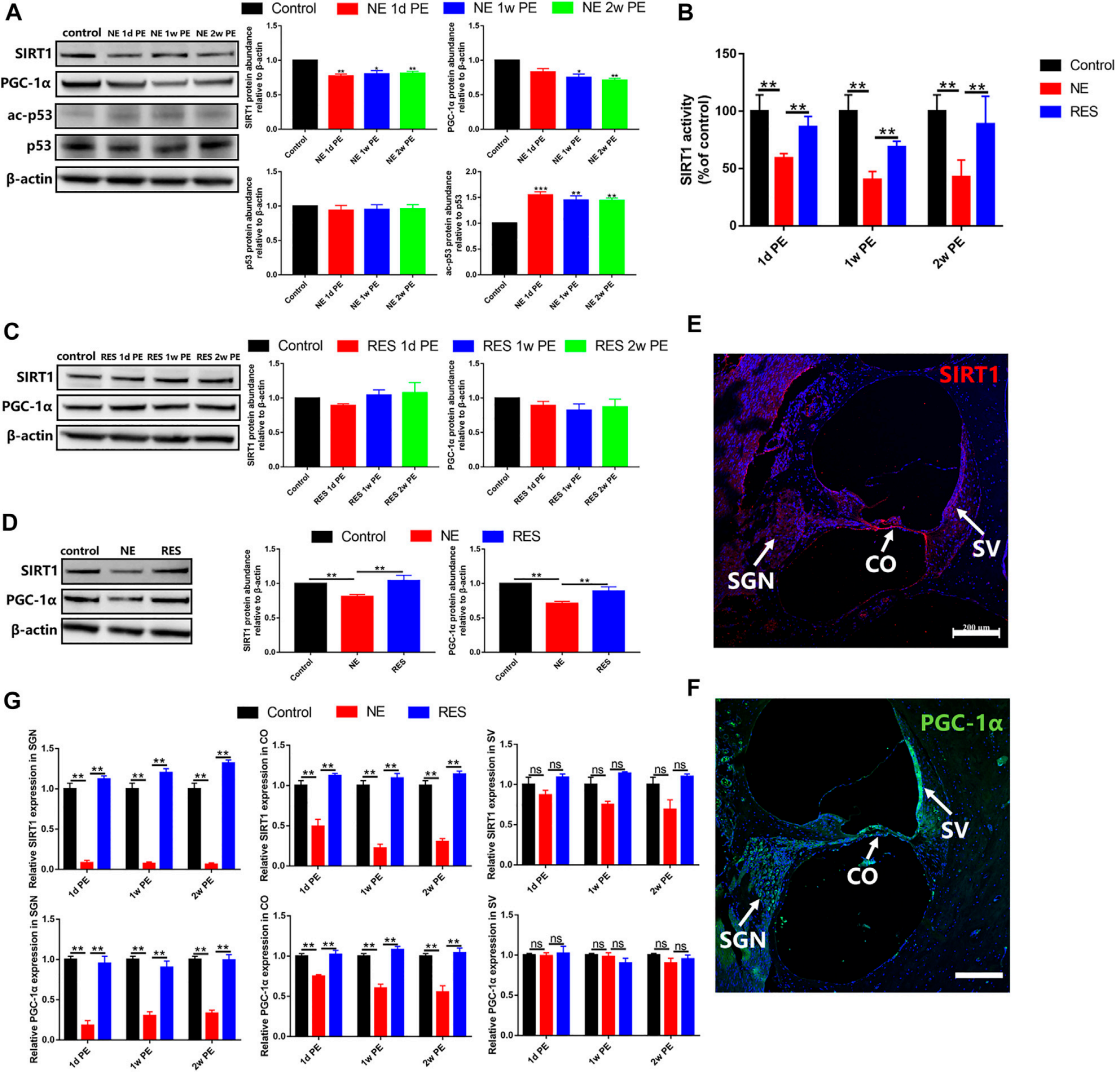
SIRT1, p53 and PGC-1α after noise exposure, the values are presented as the means ± SEM. **(A)** The protein levels of SIRT1 (130 kDa), PGC-1α (110 kDa), p53 (53 kDa) and acetylation of p53 after noise exposure in the NE group. **(B)** SIRT1 activity was measured based on an enzymatic reaction using a SIRT1 assay kit. **(C)** The protein levels of SIRT1 (130 kDa), PGC-1α (110 kDa) after noise exposure in the RES group. **(D)** The comparison among the control group, NE group and RES group at the aspect of the protein levels of SIRT1 (130 kDa), PGC-1α (110 kDa). **(E)** The expression of SIRT1 in the cochleae of guinea pigs in the control group. Scale bar = 200 µm. SV: stria vascularis, CO: Corti’s organ, SGN: spiral ganglion neuron. Red is used to mark the expression of SIRT1, and blue is used to mark the nucleus. **(F)** The expression of PGC-1α in the cochleae of guinea pigs in the control group. Scale bar = 200 µm. SV: stria vascularis, CO: Corti’s organ, SGN: spiral ganglion neuron. Green is used to mark the expression of PGC-1α, and blue is used to mark the nucleus. **(G)** The comparison among the control group, NE group and RES group at the aspect of the expression of SIRT1 and PGC-1α in cochlea by immunolabeling. SV: stria vascularis, CO: Corti’s organ, SGN: spiral ganglion neuron. Note: In **(A)**–**(D)**, n = 3 from 3 animals in each group, both cochleae of the same guinea pig as a sample. In **(G),** n = 6 from 3 animals in each group. In **(A)**, *p < 0.05, **p < 0.01, ***p < 0.001, as compared with control group.

To explore the role of the SIRT1/PGC-1α signaling pathway in NIHHL, we observed the changes of the expression and activity of SIRT1 and the changes of the expression of PGC-1α. In the NE group, the protein expression levels of SIRT1 in the 1d, 1w and 2w PE groups were significantly lower than those in the control group (Figure 4A, *p* < 0.05). Our experimental data showed that the level of SIRT1 activity in the 1d PE group decreased to a level that was significantly lower than that in the control group (Figure 4B, *p* < 0.05), and remained lower in the 2w PE group (Figure 4B, *p* < 0.05), in agreement with the observed decreases in the protein levels of SIRT1. Compared with the control group, the protein expression levels of p53 in the 1d, 1w and 2w PE groups showed no statistical differences (Figure 4A, *p* > 0.05). The formation of acetylated-p53 in the 1d, 1w and 2w PE groups increased to a level that was significantly higher than that in the control group (Figure 4A, *p* < 0.05). The protein expression level of PGC-1α in the 1d PE group exhibited a downward trend, but there were no statistically significant differences compared with the control group. In the 1w and 2w PE groups, the protein content of PGC-1α declined to a level that was significantly lower than that in the control group (Figure 4A, *p* < 0.05).

In the RES group, compared with the control group, the protein expression levels of SIRT1 in the 1d, 1w and 2w PE groups showed no statistical differences (Figure 4C, *p* > 0.05). And the protein content of PGC-1α showed no change after noise exposure, compared with the control group (Figure 4C, *p* > 0.05).

In the RES group, compared with the NE group, the protein expression levels of SIRT1 and the level of SIRT1 activity showed a significant increase (Figures 4B,D, *p* < 0.01). And the protein content of PGC-1α was significantly increased (Figure 4D, *p* < 0.01), compared with the NE group.

In the control group, SIRT1 was mainly expressed in spiral ganglion neurons, Corti’s organ and vascular stria of the cochlea (Figure 4E). In the NE group, the expression of SIRT1 decreased significantly after noise exposure, which are mainly distributed in the organ of Corti and spiral ganglion (Figure 4G, Supplementary Figure S3A). In the RES group, the expression of SIRT1 showed a significant increase in the organ of Corti and spiral ganglion (Figure 4G, Supplementary Figure S3B), compared with the NE group.

PGC-1α was mainly expressed in spiral ganglion neurons, Corti’s organ and vascular stria of the cochlea in the control group (Figure 4F). In the NE group, the expression of PGC-1α in spiral ganglion decreased most significantly, and the expression in Corti’s organ also presented a decrease (Figure 4G, Supplementary Figure S3C). In the RES group, the expression of PGC-1α showed a significant increase in the organ of Corti and spiral ganglion (Figure 4G, Supplementary Figure S3D), compared with the NE group.

### Oxidative Stress Is Involved in Noise-Induced Hidden Hearing Loss

**FIGURE 5:**
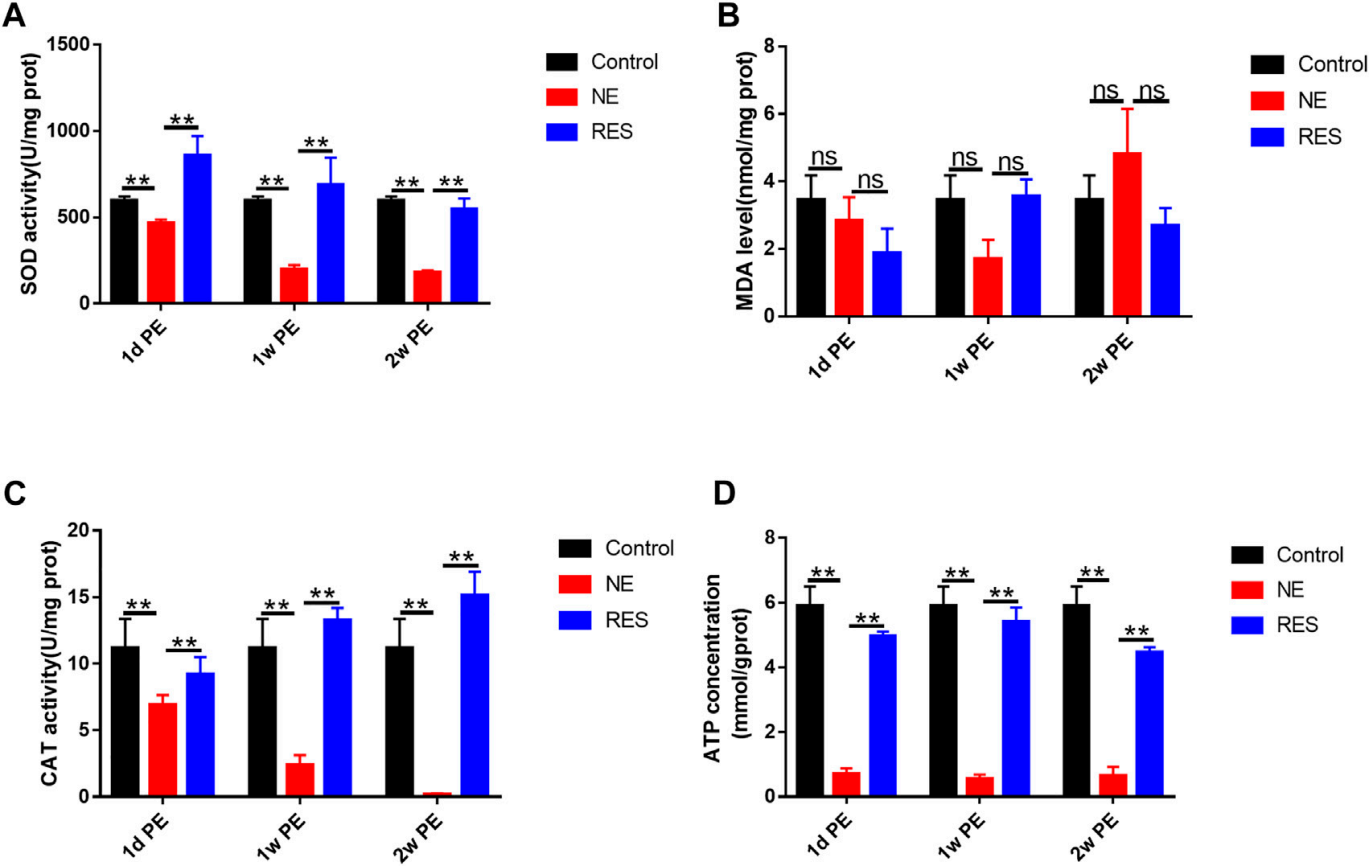
SOD activity**(A)**, MDA**(B)**, CAT**(C)** and ATP**(D)** levels in the cochleae of guinea pigs after noise exposure. The values are presented as the means ± SEM. Note: n = 3 from 3 animals in each group, both cochleae of the same guinea pig as a sample. *p < 0.05, **p < 0.01.

To explore the role of the oxidative stress in NIHHL, we observed the changes of SOD activity, MDA, CAT and ATP levels in the cochleae of guinea pigs. In the NE group, the SOD level in the control group was 597.34 ± 23.06 U/mg prot. After noise exposure, the activity decreased to 467.21 ± 19.35 U/mg prot (1d PE), with further decrease to 197.01 ± 25.84 (1w PE) and 177.88 ± 15.42 U/mg prot (2w PE; Figure 5A). Compared with the control group, the decline (*p* < 0.01) was significant. The MDA levels exhibited a range of fluctuations. Compared with the control group, the 1d, 1w and 2w PE groups showed no statistical differences (Figure 5B, *p* > 0.05). Meanwhile, CAT activity declined. Before noise exposure, the CAT activity was 11.18 ± 2.19 U/mg prot. It decreased to 6.88 ± 0.76 (1d PE; *p* < 0.05), 2.36 ± 0.76 (1w PE; *p* < 0.01), and 0.16 ± 0.08 U/mg prot (2w PE; Figure 5C, *p* < 0.01). The ATP level in the control group was 5.89 ± 0.61 mmol/g prot. After noise exposure, the level decreased to 0.71 ± 0.17 mmol/g prot (1d PE), with further decrease to 0.55 ± 0.13 (1w PE) and 0.65 ± 0.27 mmol/g prot (2w PE; Figure 5D). Compared with the control group, the decline (*p* < 0.05) was significant.

In the RES group, the level of SOD, CAT and ATP showed significant increases, compared with the NE group (Figures 5A,C,D, *p* < 0.05).

## Discussion

HHL shows normal audiometric thresholds but influences auditory neural processing and hearing acuity, particularly in noisy environments. HHL cannot be diagnosed by standard tests of auditory thresholds, such as auditory brainstem response (ABR), compound action potential (CAP) and distortion product otoacoustic emission (DPOAE) (Kohrman et al., 2020). NIHHL exhibits a TTS after noise exposure. The most mentioned mechanism of HHL is the loss of cochlear ribbon synapses between IHCs and SGNs without hair cells and SGN loss (Kujawa and Liberman, 2009). In addition, HHL could result from transient demyelination (Wan and Corfas, 2017), and heminode disruption might be the pathophysiological basis for HHL following acute demyelination. Recent studies revealed that noise-induced mild or persistent hair cell dysfunction can result in HHL (Hoben et al., 2017; Mulders et al., 2018). However, the underlying molecular mechanisms are still not precisely understood.

In previous studies, to establish an animal model of NIHHL with guinea pigs, animals were exposed for 2 h to an octave-band noise (4–8 kHz) at 106dB SPL (Lin et al., 2011), or exposed for 2 h to a broadband noise at 105 dB SPL (Liu et al., 2012; Shi et al., 2013; Song et al., 2016). Therefore, to establish an animal model of NIHHL, which is caused by high-intensity specific spectrum noise collected from a military helicopter in China, the animals were exposed to the noise at a level of 105dB SPL for 2 h. In our study, the hearing threshold of the NE group exhibited a TTS after noise exposure, but ABR wave I amplitude reduced and showed irreversible, which is in agreement with the previous studies (Lin et al., 2011; Liu et al., 2012).

Cochlear ribbon synapses are the most vulnerable elements in both noise-induced and age-related hearing loss (Liberman and Kujawa, 2017), and synaptic degeneration in noise-exposed ears exhibits loss, disorganization, and dysmorphology of synaptic ribbons (Lin et al., 2011). In previous studies (Lin et al., 2011; Liu et al., 2012; Shi et al., 2013; Song et al., 2016), after exposure to TTS inducing noise, the guinea pigs showed an immediate and irreversible loss of approximately 50% of ribbon synapses despite the absence of hair cell loss. Synaptic repair might be probable following the initial damage due to ribbon synapse plasticity. Our study provides a NIHHL model for guinea pigs and shows the dynamics of the cochlear synapse. In our study, at NE 1d PE, the basal turn decreased the most (8.27% of the control group), while the apical turn decreased to 49.97% of the control group, which is consistent with the greater reduction in the high frequency region (Lin et al., 2011; Song et al., 2016). Based on the findings of our previous study and the present study, the reasons for this include: 1) the basal turn of basilar membranes is close to the vestibular window, which has narrow ductus cochleae and poor blood circulation; 2) when low-frequency sound is conveyed from the basal turns to the apical turns and activates the maximum amplitude of the apical basilar membrane, the basal basilar membrane inevitably participates in this mechanical vibration process. Therefore, basal hair cells can also be mechanically stimulated by low-frequency sounds; 3) the hair cells of the basal turn are more sensitive to oxidative damage caused by the high expression of NOX2, which causes the OHCs of the basal turns to produce more reactive oxygen species and induce more severe damage (Qi et al., 2018). On the whole basilar membrane, the ribbons per IHC reduced to 31.03% of the control group at NE 1d PE, then returned to 42.13% of the control group at NE 1m PE, which exhibits the plasticity of ribbon synapses.

The stereocilia of cochlear hair cells can be disordered with lodging, fusion, and even falling off (Hou et al., 2003; Gilels et al., 2017) after noise exposure. In a previous study, the stereocilia of a NIHHL model exhibited slight lodging on day 14 after noise exposure (Han et al., 2020). In our study, we showed more time points after noise exposure than the previous study. The dynamics of stereocilia generally consist with changes in hearing threshold. At 1d PE and 1w PE, the hearing threshold was higher and the stereocilia were more chaotic than the control group. At 1m PE, the hearing threshold returned to the normal level, and the stereocilia were arranged orderly in most regions. The state of stereocilia was related to the auditory function, and stereocilia damage was reversible in the NIHHL model. In addition, the stereocilia of the third-row OHCs was the most chaotic, while the damage to the second-row and first-row OHCs was relatively mild. A previous study reported that this might have resulted from the location (Ren, 2002). Third-row OHCs are located in the center of the basilar membrane, where the largest vibration amplitude is located. However, the inner hair cells are located at the edge of the bony spiral lamina, which is affected by less vibration from the basilar membrane.

Oxidative stress is the result of an imbalance between oxidants and antioxidants, leading to disruption of redox signaling and control, and/or molecular damage (Jones and Sies, 2015). Han et al. observed that H_2_O_2_ increased significantly, but ATP concentration decreased significantly after noise exposure in a NIHHL model (Han et al., 2020), which indicated that cochlear injury might be induced by oxidative stress. In addition, by applying auditory metabolomics, oxidative stress has been demonstrated to be involved in noise trauma, which induces TTS, synaptopathy, and permanent hidden hearing loss (Ji et al., 2019). Superoxide dismutase (SOD) and catalase (CAT) are major antioxidant enzymes that prevent the generation of oxidation chains by scavenging the molecules responsible for generating free radicals (Singh et al., 2018). Hormesis is a dose response phenomenon characterized by a low dose stimulation and a high dose inhibition (Calabrese et al., 2007; Cornelius et al., 2013; Trovato Salinaro et al., 2018), and signaling by ROS follows the hormetic laws, whereby at low levels free radical generation regulates a wide array of physiological responses, whereas at high levels, such as in abnormal cell metabolism conditions, cell function and viability are under threat (Pennisi et al., 2017). In our study, we observed the levels of SOD, CAT, MDA and ATP dynamically, and found that the levels of SOD, CAT and ATP decreased constantly after noise exposure. At NE 2w PE, the levels of SOD, CAT and ATP were still lower than those in the control group. These results indicate a decrease in antioxidant enzymes and an imbalance between oxidants and antioxidants, and that ROS might be at a higher level and present a threat to the normal function of the cochleae.

SIRT1 has been reported to influence numerous cellular antioxidant defense mechanisms indirectly through the regulation of certain key effectors, including FOXO3a, p53, and PGC-1α(Brunet et al., 2004; Nemoto et al., 2005; Hasegawa et al., 2008; Maillet and Pervaiz, 2012). As a result, decreased intracellular ROS and increased levels of certain antioxidant proteins, such as SOD2, CAT, and glutathione peroxidase. In addition, cellular oxidative stress might contribute to dysregulation of normal SIRT1 functioning (Singh et al., 2018). The cochleae (Xiong et al., 2017; Khoshsirat et al., 2021) and auditory cortex (Chen et al., 2020) of the model of NIHL exhibited a decrease in SIRT1 expression after noise exposure, and the structure and function were aberrant. Treatment with the SIRT1 activator enhanced SIRT1 activity, reduced reactive species, promoted recovery of auditory function, and protected auditory cortex neuron cells and hair cells. Therefore, SIRT1 may play a key role in the mechanism underlying NIHL. The present study is the first to report dynamic changes in SIRT1 after HHL-induced noise exposure. In the NE group, SIRT1 expression and activity were not restored to normal level 2 weeks after noise exposure. Associating the SOD and CAT changes with SIRT1 change, we hypothesize that noise exposure might result in an imbalance between oxidants and antioxidants, which induces oxidative stress, leading to lower SIRT1 activity. It’s reported that in comparison with the normal group, the NIHL group had significantly increased mRNA and protein expression of p53 (Zhang et al., 2020a). p53 also affected the pathological process of age-related hearing loss (Zhang et al., 2020b) and drug-induced hearing loss (Ding et al., 2012). SIRT1 is reported to increase the expression of MnSOD by deacetylating p53, thus enhancing cellular antioxidant capacity (Iside et al., 2020). In our study, we explored the changes of p53 in NIHHL. Interestingly, HHL-induced noise exposure did not affect the expression of p53, whereas noise exposure significantly increased the formation of acetylated-p53, which indirectly reflected the decrease of SIRT1 activity and cellular antioxidant capacity. As a substrate of SIRT1, PGC-1α was subsequently reduced to a level lower than that of the control group, which affected reactive oxygen species detoxification and further contributed to oxidative stress. PGC-1α is viewed as a main regulator that influences the expression of several mitochondrial genes, playing a vital part in mitochondrial biogenesis (Li et al., 2017). Mitochondria are the intracellular organelles which play a significant role in the cells by metabolizing nutrients and producing ATP and responsible for various processes such as energy metabolism, generation of free radicals (Bhatti et al., 2017). The damaged mitochondria were unable to produce enough ATP to maintain the metabolism. The level of ATP declined after noise exposure and showed irreversible, which indicated the mitochondrial dysfunction and oxidative stress and reflected the decrease of PGC-1α activity. The location of SIRT1 and PGC-1α indicated that the expression in the organ of Corti and spiral ganglion decreased significantly, which consists with the finding that cochlear ribbon synapses are the most vulnerable elements.

Resveratrol is reported to have antioxidant and anti-inflammatory properties and might be used in many neurodegenerative diseases and metabolic disorders (Chung et al., 2012; Seo et al., 2012; Cottart et al., 2014). Although the mechanisms of resveratrol’s protective effects are not fully revealed, several studies have showed that resveratrol augments NAD levels and SIRT1 activity and is considered a potent SIRT1 agonist (Park et al., 2012; Price et al., 2012; Sin et al., 2014). In the animal models of NIHL (Xiong et al., 2017) and AIHL (Xiong et al., 2019), resveratrol could attenuate the injury and promote the recovery of hearing by enhancing cochlear SIRT1 activity. In our study, we also chose resveratrol as a SIRT1 agonist to explore the role of the SIRT1/PGC-1α signaling pathway in NIHHL. With resveratrol treatment, SIRT1 expression and activity showed a significant increase after noise exposure, compared with the NE group. The PGC-1α and antioxidant proteins also increased after noise exposure, compared with the NE group. As a result, the damage to the ribbon synapses and the stereocilia were attenuated. The auditory function, especially ABR wave I amplitudes, was also promoted. These results further indicate the involvement of the SIRT1/PGC-1α signaling pathway in noise-induced hidden hearing loss.

Our labs have explored the involvement of the SIRT1/PGC-1α signaling pathway in NIHL before, finding that noise-induced auditory cortex damage may involve down-regulation of the SIRT1/PGC-1a signaling pathway, which is reversed by ginsenoside Rd treatment through enhancement of auditory cortex SIRT1 activity and reduction of oxidative stress (Chen et al., 2020). It’s also reported that the SIRT1/PGC-1α signaling pathway is involved in age-related hearing loss (Tian et al., 2014; Xue et al., 2016; Shen et al., 2018; Hao et al., 2019), and the downregulated SIRT/PGC-1α increases the incidence of age-related hearing loss via promoting the apoptosis of cochlear hair cells. The present study is the first to explore the involvement of the SIRT1/PGC-1α signaling pathway in NIHHL and may shed light on the underlying molecular mechanisms.

## Conclusion

In summary, the present study established a NIHHL model after noise exposure and explored the dynamic changes in the synapses and the morphologic variations of the cochlea stereocilia. In addition, we studied the changes in the SIRT1/PGC-1α signaling pathway and the major antioxidant enzymes and concluded that the SIRT1/PGC-1α signaling pathway and oxidative stress might mutually affect and contribute to the pathological process of NIHHL.

## Data Availability

The original contributions presented in the study are included in the article/Supplementary Material, further inquiries can be directed to the corresponding authors.

## Supplementary Material

Supplementary Figure S1 The alterations of 20–70 dB SPL ABR wave Ⅰ amplitude of guinea pigs in each group after 105dB SPL military helicopter noise exposure (µV). Note: n = 12 ears from 6 animals in each group. *P < 0.05, **P < 0.01.

Supplementary Figure S2 Hair cells survival after noise exposure. The values are presented as the means ± SEM. n = 24 in each group.

Supplementary Figure S3 The expression of SIRT1 and PGC-1α in the cochleae of guinea pigs after noise exposure. **(A)** The expression of SIRT1 in the NE group after noise exposure. **(B)** The expression of SIRT1 in the RES group after noise exposure. **(C)** The expression of PGC-1α in the NE group after noise exposure. **(D)** The expression of PGC-1α in the RES group after noise exposure. Scale bar = 200 µm. SV: stria vascularis, CO: Corti’s organ, SGN: spiral ganglion neuron.
