# Supplementary figures and images for: Involvement of the SIRT1/PGC-1α Signaling Pathway in Noise-Induced Hidden Hearing Loss
Source: Front Physiol. 2022 May 10;13:798395. doi: 10.3389/fphys.2022.798395 (PMC9127058; doi:10.3389/fphys.2022.798395)

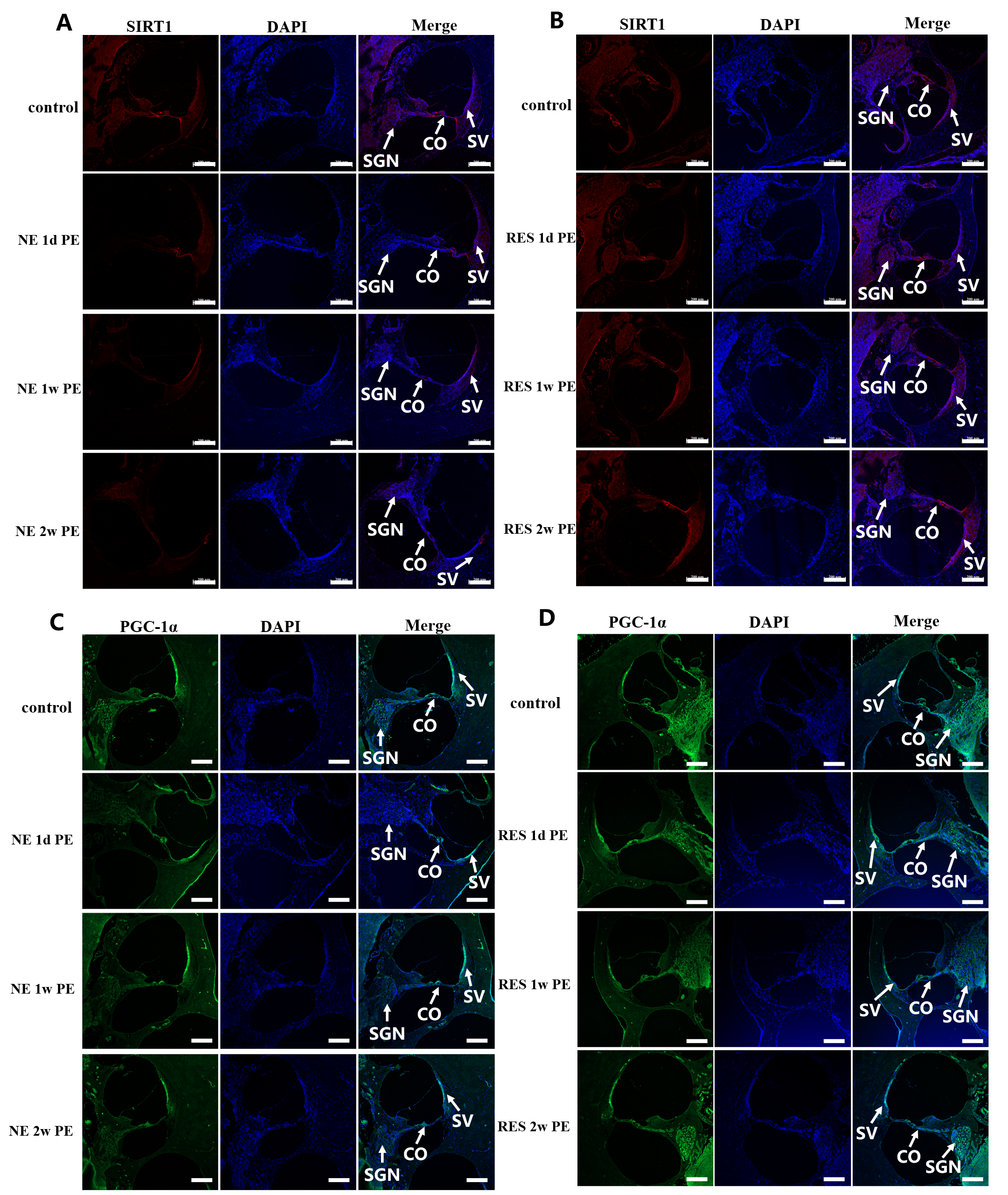

Supplement: Supplementary file 1 [file Image3.tif]

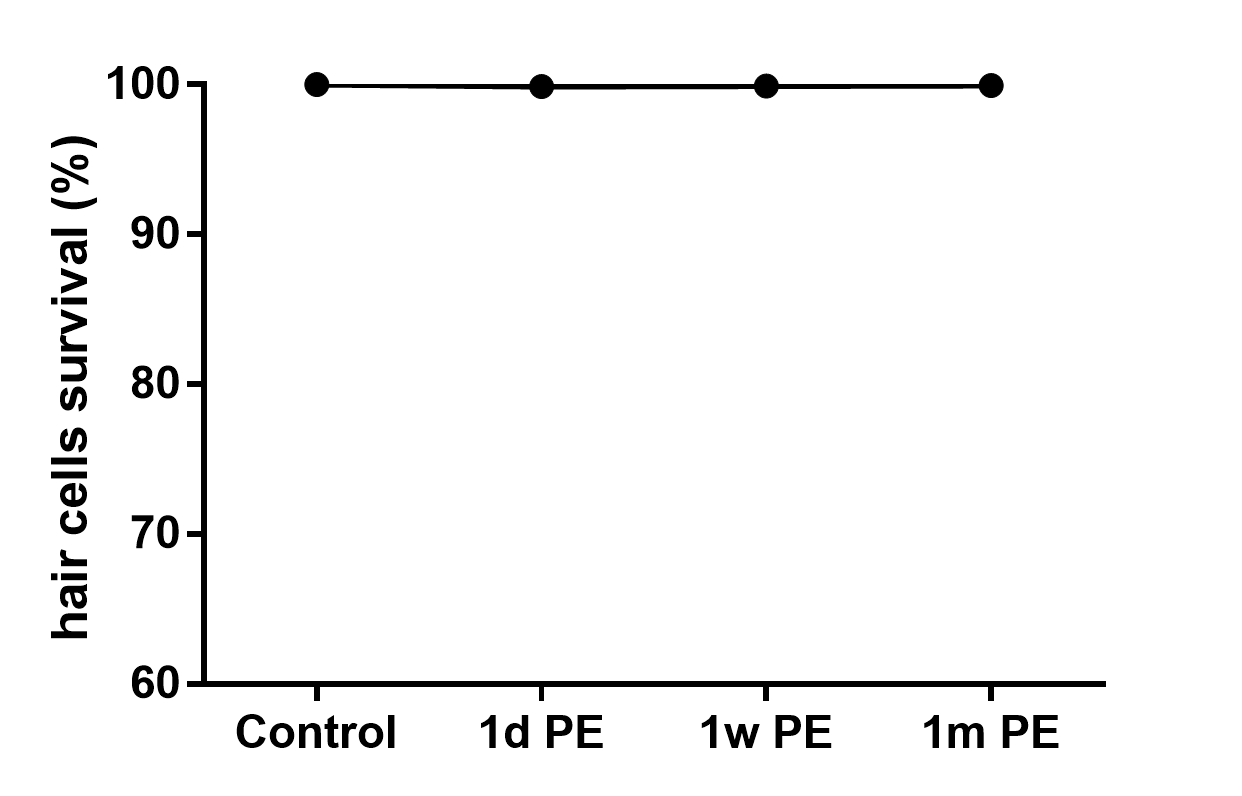

Supplement: Supplementary file 2 [file Image2.tif]

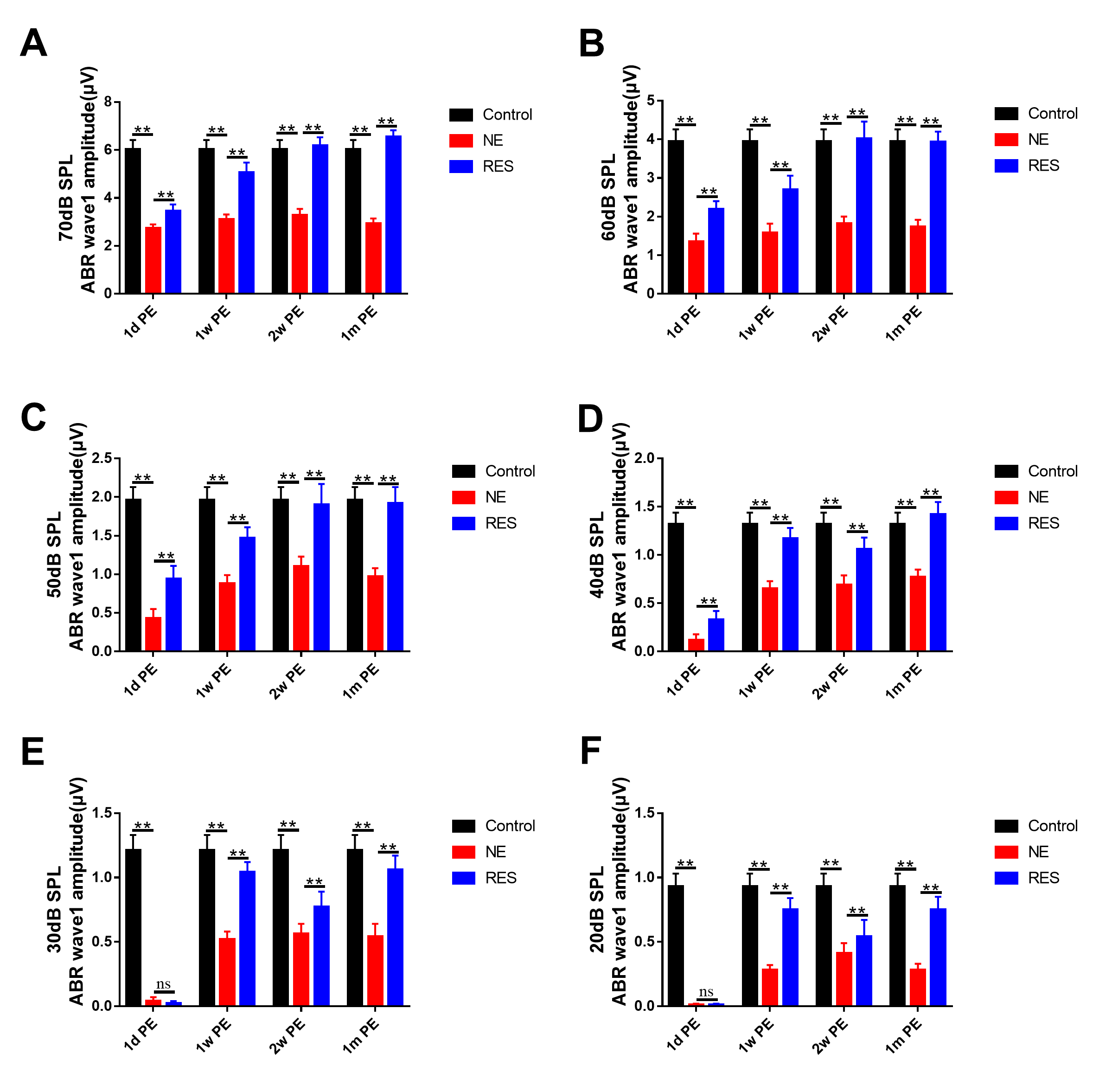

Supplement: Supplementary file 3 [file Image1.tif]
